# Different experimental multiple trauma models induce comparable inflammation and organ injury

**DOI:** 10.1038/s41598-020-76499-z

**Published:** 2020-11-19

**Authors:** Borna Relja, Bing Yang, Katrin Bundkirchen, Baolin Xu, Kernt Köhler, Claudia Neunaber

**Affiliations:** 1 Department of Radiology and Nuclear Medicine, Experimental Radiology, Otto-von-Guericke University, Magdeburg, Germany; 2 Trauma Department, Hannover Medical School, Hannover, Germany; 3 Department of Trauma, Hand and Reconstructive Surgery, Goethe University Frankfurt, Frankfurt am Main, Germany; 4 Institute of Veterinary Pathology, Justus Liebig University, Giessen, Germany

**Keywords:** Experimental models of disease, Preclinical research, Cytokines, Inflammation

## Abstract

Multiple injuries appear to be a decisive factor for experimental polytrauma. Therefore, our aim was to compare the inflammatory response and organ damage of five different monotrauma with three multiple trauma models. For this, mice were randomly assigned to 10 groups: Healthy control (Ctrl), Sham, hemorrhagic shock (HS), thoracic trauma (TxT), osteotomy with external fixation (Fx), bilateral soft tissue trauma (bsTT) or laparotomy (Lap); polytrauma I (PT I, TxT + HS + Fx), PT II (TxT + HS + Fx + Lap) and one multi-trauma group (MT, TxT + HS + bsTT + Lap). The inflammatory response and organ damage were quantified at 6 h by analyses of IL-6, IL-1β, IL-10, CXCL1, SAA1, HMGB1 and organ injury. Systemic IL-6 increased in all mono and multiple trauma groups, while CXCL1 increased only in HS, PT I, PT II and MT vs. control. Local inflammatory response was most prominent in HS, PT I, PT II and MT in the liver. Infiltration of inflammatory cells into lung and liver was significant in all multiple trauma groups vs. controls. Hepatic and pulmonary injury was prominent in HS, PT I, PT II and MT groups. These experimental multiple trauma models closely mimic the early post-traumatic inflammatory response in human. Though, the choice of read-out parameters is very important for therapeutic immune modulatory approaches.

## Introduction

Trauma is one of the leading causes of death in all age groups worldwide^1^. Despite significant improvements in timely surgical and critical strategies as well as in the intensive care of multiply traumatized patients, both mortality and disability rates still remain high^2^. Due to the heterogeneity of trauma, its complex injury patterns, and a wide variety of therapeutic options, the collection of reliable and valid data concerning the posttraumatic pathophysiology, and subsequently the treatment options is challenging. Epidemiological data from 2016 to 2018 of the DGU Trauma Registry show that 81% of traumatized patients suffer from serious injuries. Of these patients 45.4% suffered from serious injuries of the head, 45.8% of serious injuries of the thorax and 28.1% of serious injuries of the extremities^3^. Furthermore, the most preventable cause of death constitutes the uncontrolled bleeding or hemorrhagic shock (HS)^4^. Even though the certain injury type predominantly causes mortality, notably the combination of multiple injuries aggravates the outcome after trauma^5^. Patients who initially survive certain trauma types and injury pattern are at high risk for developing post-injury complications with organ dysfunctions or infections, which are caused by the complex immune response to trauma^6,7^.

There have been numerous attempts to develop multiple trauma models mimicking the post-injury situation, to evolve therapeutic interventions for improving post-traumatic outcomes. And although several preclinical models have provided promising results, efforts at interventional strategies in clinical reality failed^8–10^. Consequently, the question about the suitability of the used multiple injury models to represent the clinical situation are currently under discussion^9,11–14^. With regard to trauma, an anatomical scoring system the so-called Injury Severity Score (ISS) above 15 is applied to define the polytrauma or “severely injured” or “multiple traumatized” patients^15,16^. Yet the definition of the ISS is vulnerable, since it does not always reflect the physiological course after injury^17,18^. Therefore, additional criteria such as laparotomy, severe shock, admission to the intensive care unit, a systemic inflammatory response syndrome^19^, or combining the concept of different injury patterns with the addition of physiological responses are meanwhile defining the new “Berlin definition” of polytraumatized patients^20^.

The most common in vivo trauma models combine mainly two insults to better imitate the human conditions^9,21–26^. However, considering the definition of polytrauma according to the ISS or the new Berlin Definition, an experimental in vivo model combining two insults does not fully recapitulate the multicompartmental clinical situation. In an effort to better replicate the human trauma, Gentile et al. introduced a non-lethal murine multiple trauma model with an ISS of 18 consisting of hemorrhagic shock, laparotomy with cecectomy, and femur fracture with muscle tissue damage^27^. Huber-Lang et al. considered the significance of TBI and established a murine clinically relevant experimental polytrauma model of a blunt chest trauma, head injury, femur fracture and soft tissue injury^28,29^. Recently, Mira et al. confirmed the murine model of polytrauma of Gentile et al. showing that their model produces an equivalent ISS associated with adverse outcomes in humans, and may better recapitulate the human leukocyte, cytokine, transcriptomic, and overall inflammatory response after polytrauma^9,27^. The authors discuss that a further increase in the ISS would likely lead to a superior model that may better represent the human trauma.

In general, little is known about the relevance of different monotrauma types which are included in polytrauma modeling, or about the importance of the ISS for the complex post-traumatic immune response in experimental in vivo polytrauma modelling. To further clarify the specific roles of diverse monotrauma as well as the relevance of the injury severity, we compared the post-traumatic inflammatory response and tissue damage in five different monotrauma to two polytrauma models (ISS ≥ 16) and one multiple trauma model (ISS < 16), to provide more information about the suitability of each model to reflect the clinical situation in human.

## Materials and methods

### Animals

All animal experiments were performed with permission of the Veterinary Institute for Animal Welfare of the Lower Saxony State Office for Consumer Protection and Food Safety, Germany (Approval No. 33.12-42502-04-13/1323), as well as in accordance with the German Animal Welfare Legislation, and approved by the local institutional animal care and research advisory committee of the Hannover Medical School. In this study, 12 week old male C57BL/6NCrl mice (Charles River Laboratories, Sulzfeld, Germany) were fed and housed under standardized conditions^24,29,30^.

Anesthesia was administered by inhalational isoflurane (Baxter Deutschland GmbH, Unterschleißheim, Germany) vaporization and 1% prilocainhydrochlorid (Xylonest, AstraZeneca GmbH, Wedel, Germany) was used for local anesthesia. Additionally, 1 mg/kg body weight butorphanol (Torbugesic, Zoetis Deutschland GmbH, Berlin, Germany) combined with 5 mg/kg body weight carprofen (Rimadyl, Zoetis Deutschland GmbH, Berlin, Germany) were applied subcutaneously^30^. As postoperative analgesia 0.8 mg/mL Novaminsulfon Lichtenstein 500 mg (Zentiva Pharma GmbH, Frankfurt am Main, Germany) was added to the drinking water as described before^30^. Warming lamps and heat pads were used to keep the body temperature stable during the operation. The animals were allowed to awake immediately upon intervention and had free access to food and water.

### Group allocation and experimental model

The experimental setting was described before^30^. Prior to experimental procedures randomization of mice were performed to one of ten groups. Briefly, we included one control group without interventions (control, Ctrl), and a Sham group undergoing catheterization and ligation of the femoral artery without blood loss and reperfusion (Sham). The five single monotrauma groups consists of bilateral soft tissue trauma (bsTT), hemorrhagic shock (HS), laparotomy (Lap) undergoing abdominal trauma, osteotomy with external fixation (Fx), and thoracic trauma (TxT). Furthermore, we included three multiple trauma groups: The polytrauma (PT) I group consists of combinatory Fx, HS, and TxT and PT II group consisting of Fx, HS, TxT and Lap. The multi-trauma (MT) group received HS, Lap, TxT, and bsTT was included instead of Fx. The injury severity was calculated as described before^29–31^ in all multiple trauma groups according to the ISS system. The group allocation with animal number is shown in Table 1. Six hours after completion of the trauma induction animals were sacrificed under deep anesthesia with isoflurane.

**Table 1:** Group allocation. 153 mice were subdivided in ten groups.

Group	Treatment procedure	Number [n]
Ctrl	Healthy animals without intervention	15
Sham	Catheter placement	15
Fx	Osteotomy + external fixation	16
Lap	Midline Laparotomy (2 cm)	16
HS	Hemorrhagic shock & reperfusion with Ringer's solution	15
TxT	Thoracic trauma	15
bsTT	Bilateral soft tissue trauma (lower leg)	16
PT I	TxT + HS + Fx	18
PT II	TxT + HS + Fx + Lap	18
MT	TxT + HS + Lap + bsTT	18

The total number of animals in each group is indicated. *bsTT*: bilateral soft tissue trauma; *Ctrl*: no interventions; *Fx*: osteotomy with external fixation; *HS*: hemorrhagic shock; *Lap*: midline laparotomy; *MT*: multi-trauma (TxT + HS + Lap + bsTT); *PT*: polytrauma (PT I: TxT + HS + Fx and PT II: TxT + HS + Fx + Lap); *Sham*: surgical procedures without trauma; TxT: thoracic trauma.

Osteotomy with external fixation (Fx) was induced as described before^30^. Here, a longitudinal approach of the skin was performed followed by the opening of the tensor fasciae latae. To expose the full length of the femur, the vastus lateralis biceps femoris muscles were split bluntly. Afterwards, MouseExFix simple L 100% external fixator system (RISystem AG, Davos, Switzerland) was attached to the femur^24,30,25^. Subsequent osteotomy was induced by a 0.44 mm Gigli wire saw (RISystem AG) at the middle of the femur. Afterwards, the f*ascia lata* was closed with continuous and the skin with interrupted sutures.

As described before laparotomy (Lap) was performed in a supine position^30^. 1 ml of preheated 0.9% sodium chloride solution was provided into the cavity. Finally, the abdominal muscle was closed with continuous and the skin with interrupted sutures.

Induction of hemorrhagic shock (HS) was performed as described previously^30^. First, the femoral artery was exposed and cannulated with polyethylene tubing (Becton Dickinson and Company, Sparks, MD, USA). Afterwards, animals were bled from a physiologic mean arterial blood pressure^25^ via the catheter to a mean arterial blood pressure of 35 ± 5 mm Hg for induction and maintenance of HS. Blood pressure was maintained for 90 min and constantly monitored during bleeding by a measuring cell (FMI TBD-1222, Föhr Medical Instruments GmbH, Seeheim, Germany) and a measuring amplifier (MIO-0501 DC, Föhr Medical Instruments GmbH). After HS, resuscitation with four times the shed blood volume with preheated Ringer’s solution (37.5 °C) (Berlin-Chemie AG, Berlin, Germany) was performed over 30 min. At the end of the reperfusion process the average blood pressure was 60 mm Hg. Then, the catheter was removed, and the vessels were occluded. Finally, the incision was closed with interrupted sutures.

Bilateral soft tissue trauma (bsTT) was modified as published before^32,33^. Induction of bsTT was induced with the same device which was used for thoracic trauma^30^.

Blunt thoracic trauma (TxT) was induced in a supine position under anesthesia by a 300 g aluminum weight falling on the chest of the mice as described previously^24^.

### Blood and organ sampling

Blood was obtained via cardiac puncture by a heparinized syringe at 6 h after completion of the trauma. Plasma was isolated from blood samples after centrifugation at 2500×*g* for five minutes at room temperature (Eppendorf 3200, Hamburg, Germany) and then stored at − 80 °C for later analyses of IL-6, IL-1 β, SAA1 and CXCL1 using the specific Mouse DuoSet ELISA Kits of R&D Systems according to the manufacturer’s instructions (Wiesbaden-Nordenstadt, Germany). ELISA was performed using an Infinite M200 microplate reader (Tecan, Männedorf, Switzerland).

After blood withdrawal liver and lungs were removed. One lung lobe was snap-frozen using liquid nitrogen and stored at − 80 °C until further analyses. The other lung lobe was filled with 4% formalin for overnight fixation and histomorphological analyses as described below. The left liver lobe was ligated, removed, snap-frozen in liquid nitrogen and stored at − 80 °C until further analyses. The remaining liver was flushed with 20 mL 10% buffered formalin solution and removed for further handling for (immuno)histology as described below.

### RNA isolation and reverse transcription semi-quantitative polymerase chain reaction (RT-qPCR)

Snap frozen specimens from liver and lung were stored at − 80 °C after sacrifice. RNA isolation was performed following the manufacturer's instructions using the RNeasy-system (Qiagen, Hilden, Germany). To remove residual DNA the RNase free DNase kit (Qiagen, Hilden, Germany) was used. The quantity and quality of RNA were determined using the NanoDrop ND-1000 device (Nano Drop Technologies, Wilmington, USA). The Affinity Script QPCR-cDNA synthesis kit (Stratagene, La Jolla, CA, USA) was used according to manufacturer’s instructions for cDNA synthesis. Gene expressions of Hmgb1, Il-6 and Mmp9 were quantified by using specific primer for mouse Hmgb1 (NM_031168, UniGene#: Mm.1019, Cat#: PPM03015A), mouse Il6 (NM_031168, UniGene#: Mm.1019, Cat#: PPM03015A), mouse Mmp9 (NM_031168, UniGene#: Mm.1019, Cat#: PPM03015A), and mouse Gapdh (NM_008084, UniGene#: Mm.343110, Cat#: PPM02946E, SABiosciences, SuperArray, Frederick, MD, USA) as housekeeping gene (control). The total volume of the PCR reaction was 25 µl with RT2 SYBR Green/Rox qPCR Master mix (SABiosciences) according to the manufacturer’s instructions. The PCR reaction was performed on a Stratagene MX3005p QPCR system (Stratagene) and consists of an initial denaturation step at 95 °C for 10 min, followed by 40 cycles of denaturation at 95 °C for 15 s, and annealing/extension at 60 °C for 60 s. The relative gene expression of each target gene normalized to Gapdh was calculated by using the 2-ΔΔCT method (comparative threshold-cycle (CT) method).

### Quantification of CXCL1 expression levels in liver and lungs

Liver and lung tissue were homogenized in protein lysis buffer at 4 °C, followed by centrifugation for 30 min at 4 °C at 20,000×*g*. Supernatants were stored at − 80 °C for later analyses of protein concentration of CXCL1 using a Mouse CXCL1/KC DuoSet ELISA kit of R&D Systems according to manufacturer’s instructions (Wiesbaden-Nordenstadt, Germany). ELISA was performed using the Infinite M200 microplate reader (Tecan, Männedorf, Switzerland).

### Immunohistological staining of IL-6, IL-10 and HMGB1

For the determination of the IL-6, IL-10 and HMGB1 expression in liver and lungs, paraffin-embedded sections were deparaffinized, rehydrated, and stained with antibodies as described earlier^21^. First, antigen retrieval was performed using R-universal solution (Aptum Biologics) under steam atmosphere for 20 min (Retriever 2010) and then, hydrogen peroxide was applied for blocking the endogenous peroxidase activity (Peroxidase UltraVision Block). Anti-IL-6 antibody (ab208113), Anti-IL-10 (ab192271) and Anti-HMGB1 (ab18256, all abcam, diluted as suggested by the manufacturer) in Antibody Diluent with Background Reducing Components (Dako) were used as primary antibodies for 1 h at room temperature. The secondary horseradish peroxidase-linked antibody (rat or rabbit, respectively, Histofine Simple Stain, Nichirei Biosciences Inc.) were applied to detect the corresponding primary antibody and 3-amino-9-ethylcarbazol (AEC, DCS Innovative Diagnostik-Systeme, Hamburg) was used as substrate to detect specific binding. In the end, hematoxylin was used for counterstaining of the samples.

### Examination of organ damage

Specimens were fixated in 4% formalin overnight and embedded in paraffin. For subsequent staining with hematoxylin–eosin (HE) sectioning of 3 µm samples was performed. An independent examiner (KK) determined histological tissue damage of HE-stained sections of the various experimental groups in a blinded manner as described before^40,41^. Lung sections were examined for interstitial neutrophilic infiltration and for interstitial thickness (alveolar space), whereas liver sections were evaluated for infiltration with inflammatory cells (neutrophilic granulocytes) via the Ly6G staining (Anti-Ly6g antibody [RB6-8C5] (ab25377)) and necrosis.

### Statistical analysis

Statistical analysis was performed using GraphPad Prism 6 (GraphPad Software, Inc., San Diego, CA). Based on the histogram and Shapiro–Wilk test, the non-parametric Kruskal–Wallis test, which does not assume a normal distribution of the residuals, followed by Dunn’s post hoc test for the correction of multiple comparisons were applied. Results were expressed as median and minimum to maximum. A p value of less than 0.05 was considered to be statistically significant.

## Results

### Systemic IL-6, IL-1β, SAA1 and CXCL1 concentrations

**Figure 1:**
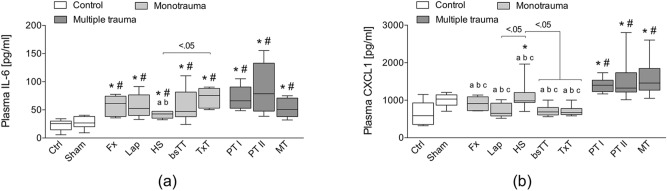
(**a**) Systemic interleukin (IL)-6 and (**b**) systemic chemokine (C-X-C motif) ligand (CXCL)1 levels are shown. *bsTT* bilateral soft tissue trauma, *Ctrl* no intervention, *Fx* osteotomy with external fixation, *HS* hemorrhagic shock, *Lap* midline laparotomy, *MT* multi-trauma (TxT + HS + Lap + bsTT), *PT* polytrauma (PT I: TxT + HS + Fx and PT II: TxT + HS + Fx + Lap). *Sham* surgical procedures without trauma, *TxT* thoracic trauma. Six hours after intervention, the animals were euthanized for sampling. p < 0.05 vs. indicated or * vs. Ctrl; # vs. Sham; a vs. PT I; b vs. PT II; c vs. MT.

At six hours after trauma, systemic IL-6 levels were significantly increased in all trauma groups compared to Ctrl or Sham, respectively (p < 0.05, Fig. 1a). While the IL-6 level was significantly increased in the TxT group compared to the HS group (p < 0.05), no significant differences between the other trauma groups were observed (Fig. 1a). Furthermore, IL-6 was significantly enhanced in both PT groups compared to the HS group (p < 0.05; Fig. 1a).

The level of circulating CXCL1 were significantly increased in both PT groups and in the MT group compared to Ctrl, Sham, Fx, Lap, HS, bsTT and TxT groups, respectively (p < 0.05, Fig. 1b). CXCL1 was significantly enhanced in the HS group compared to Ctrl, Lap, bsTT or TxT group, respectively (p < 0.05, Fig. 1b).

No significant differences between the groups were observed for IL-1β or SAA1 (data not shown).

### Quantification of hepatic and pulmonary gene expression of proinflammatory mediators

**Figure 2:**
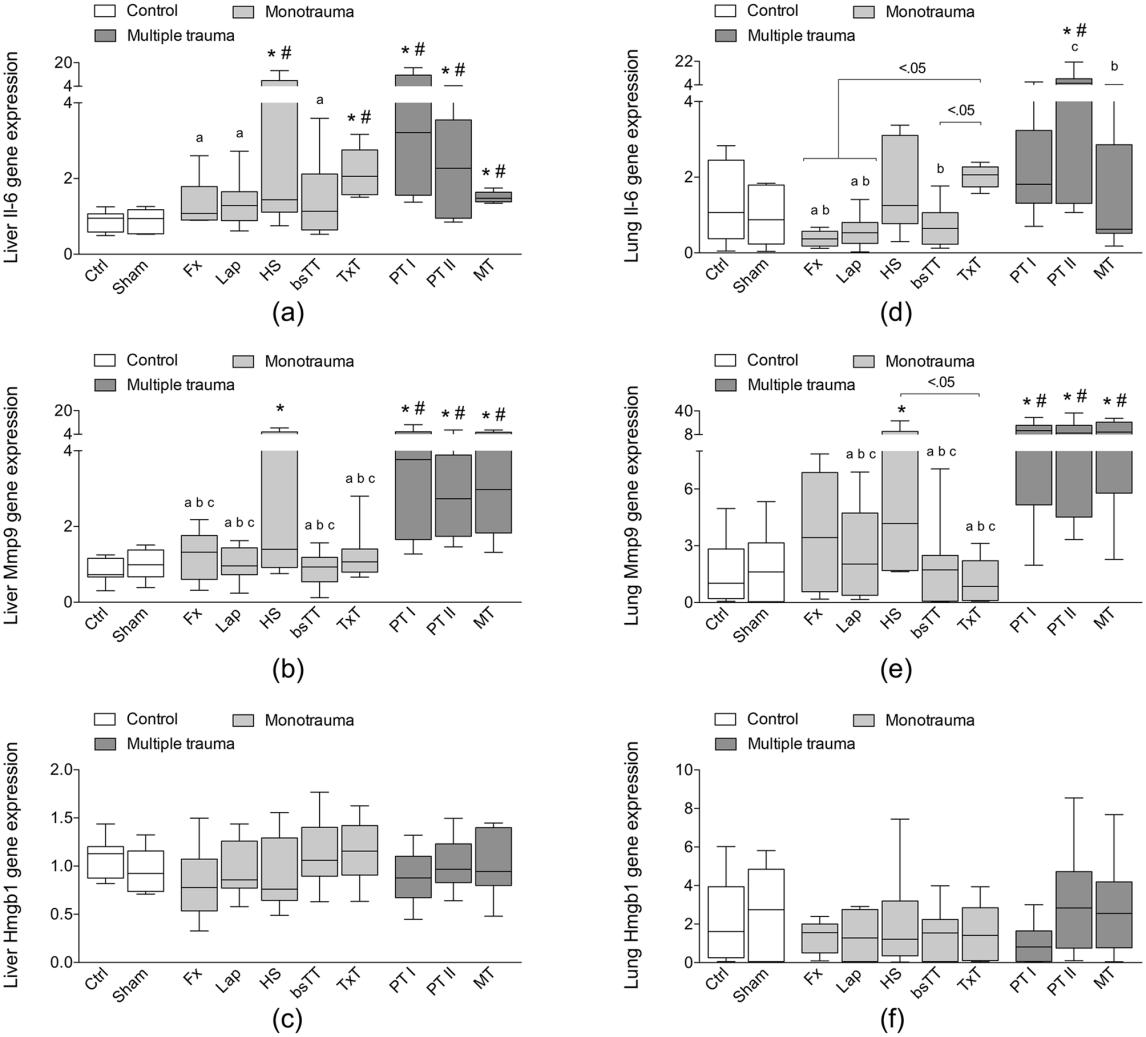
Hepatic (**a**–**c**) or pulmonary (**d**–**f**) gene expression of interleukin (Il)-6 (**a**, **d**), matrix metallopeptidase (Mmp)9 (b and e) and high mobility group box protein (Hmgb)1 (**c**, **f**) is shown. *bsTT* bilateral soft tissue trauma, *Ctrl* no intervention, *Fx* osteotomy with external fixation, *HS* hemorrhagic shock, *Lap* midline laparotomy, *MT* multi-trauma (TxT + HS + Lap + bsTT), *PT* polytrauma (PT I: TxT + HS + Fx and PT II: TxT + HS + Fx + Lap). *Sham* surgical procedures without trauma, *TxT* thoracic trauma. Six hours after intervention the animals were euthanized for sampling. p < 0.05 vs. indicated or * vs. Ctrl; # vs. Sham; a vs. PT I; b vs. PT II; c vs. MT.

At six hours after trauma, gene expression of Il-6 in the liver significantly increased in the HS and in the TxT group as well as in all multiple trauma groups (PT I, PT II, and MT) compared to Ctrl or Sham (p < 0.5, Fig. 2a). Furthermore, Il-6 gene expression was significantly enhanced in the PT I group compared to Fx, Lap and bsTT groups (p < 0.05; Fig. 2a).

Hepatic gene expression of Mmp9 significantly increased in the HS group as well as in all three multiple trauma groups compared to the Ctrl (p < 0.05, Fig. 2b). Mmp9 gene expression in the liver was significantly enhanced in all three multiple trauma groups compared to Fx, Lap, TxT and TxT group (p < 0.05, Fig. 2b).

No significant differences with regard to the Hmgb1 gene expression were observed among the groups in the liver (Fig. 2c).

Il-6 gene expression in the lung significantly increased in the PT II group compared to Ctrl or Sham (p < 0.5, Fig. 2d). The pulmonary gene expression of Il-6 in the PT I group was significantly enhanced compared to Fx and Lap (p < 0.05; Fig. 2d), while in the PT II group a significant increase was observed compared to Fx, Lap as well as bsTT groups (p < 0.05, Fig. 2d). TxT group had a significantly increased Il-6 gene expression in the lung compared to Fx, Lap and bsTT groups (p < 0.05, Fig. 2d). Among the multiple trauma groups, a significantly decreased Il-6 gene expression in the lungs from MT group compared to the PT II group was detected (p < 0.05, Fig. 2d).

Pulmonary gene expression of Mmp9 significantly increased in the HS group as well as in all three multiple trauma groups compared to the Ctrl group (p < 0.05, Fig. 2e). Furthermore, all three multiple trauma groups had significantly enhanced Mmp9 expression compared to either Ctrl, Sham, Lap, bsTT or TxT groups (p < 0.05, Fig. 2e).

No significant differences with regard to the Hmgb1 gene expression in the lung were observed among the groups (Fig. 2f).

### CXCL1 protein expression in liver and lung tissue

**Figure 3:**
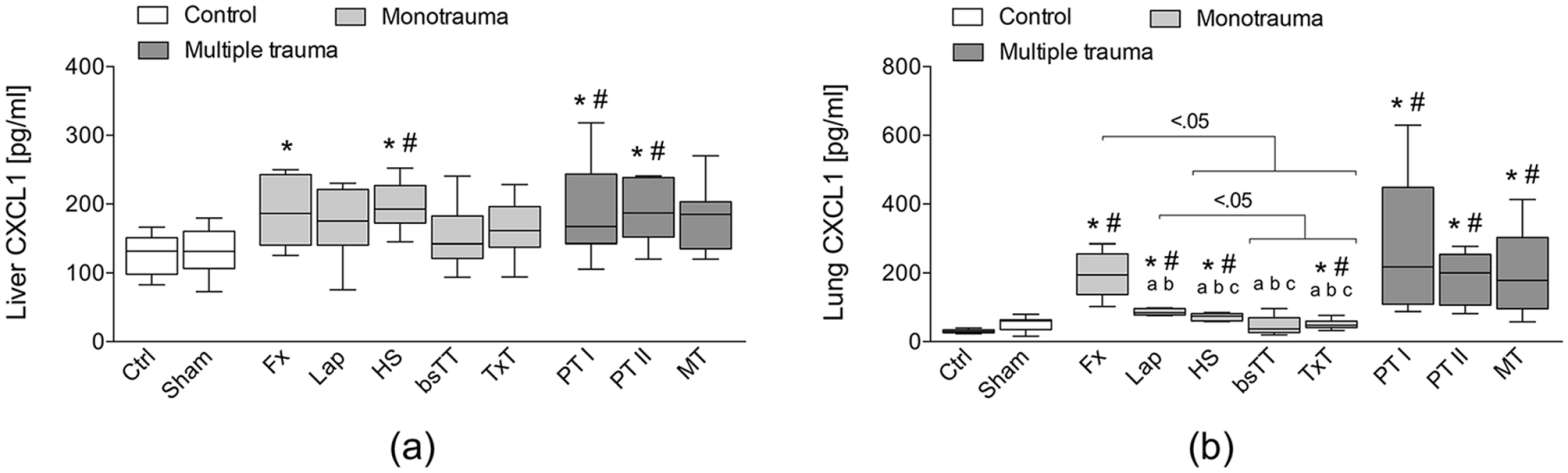
(**a**) Hepatic or (**b**) pulmonary protein expression of chemokine (C-X-C motif) ligand (CXCL)1. *bsTT* bilateral soft tissue trauma, *Ctrl* no intervention, *Fx* osteotomy with external fixation, *HS* hemorrhagic shock, *Lap* midline laparotomy, *MT* multi-trauma (TxT + HS + Lap + bsTT), *PT* polytrauma (PT I: TxT + HS + Fx and PT II: TxT + HS + Fx + Lap), *Sham* surgical procedures without trauma, *TxT* thoracic trauma. Six hours after intervention, the animals were euthanized for sampling. p < 0.05 vs. indicated or * vs. Ctrl; # vs. Sham; a vs. PT I; b vs. PT II; c vs. MT.

Hepatic CXCL1 protein expression was significantly increased in the HS group as well as in PT I and PT II groups, respectively, compared to Ctrl or Sham group (p < 0.05, Fig. 3a), while the increase in CXCL1 protein expression in the Fx group was significant compared to the Ctrl (p < 0.05, Fig. 3a).

Except in the bsTT group, the CXCL1 protein expression in the lung was significantly increased in all monotrauma as well as multiple trauma groups compared to Ctrl or Sham group (p < 0.05, Fig. 3b). Furthermore, lung CXCL1 levels were significantly increased in Fx group compared to HS, bsTT and TxT, as well as CXCL1 levels of Lap were significantly increased to bsTT and TxT. Also, CXCL1 levels were significantly increased in both PT groups compared to Lap, HS, bsTT and TxT groups, respectively (p < 0.05, Fig. 3b). In the MT group, the CXCL1 protein expression in the lung was significantly enhanced compared to the HS, bsTT or TxT group, respectively (p < 0.05, Fig. 3b).

### Immunohistological evaluation of IL-6, IL-10 and HMGB1 expression in liver or lungs

**Figure 4:**
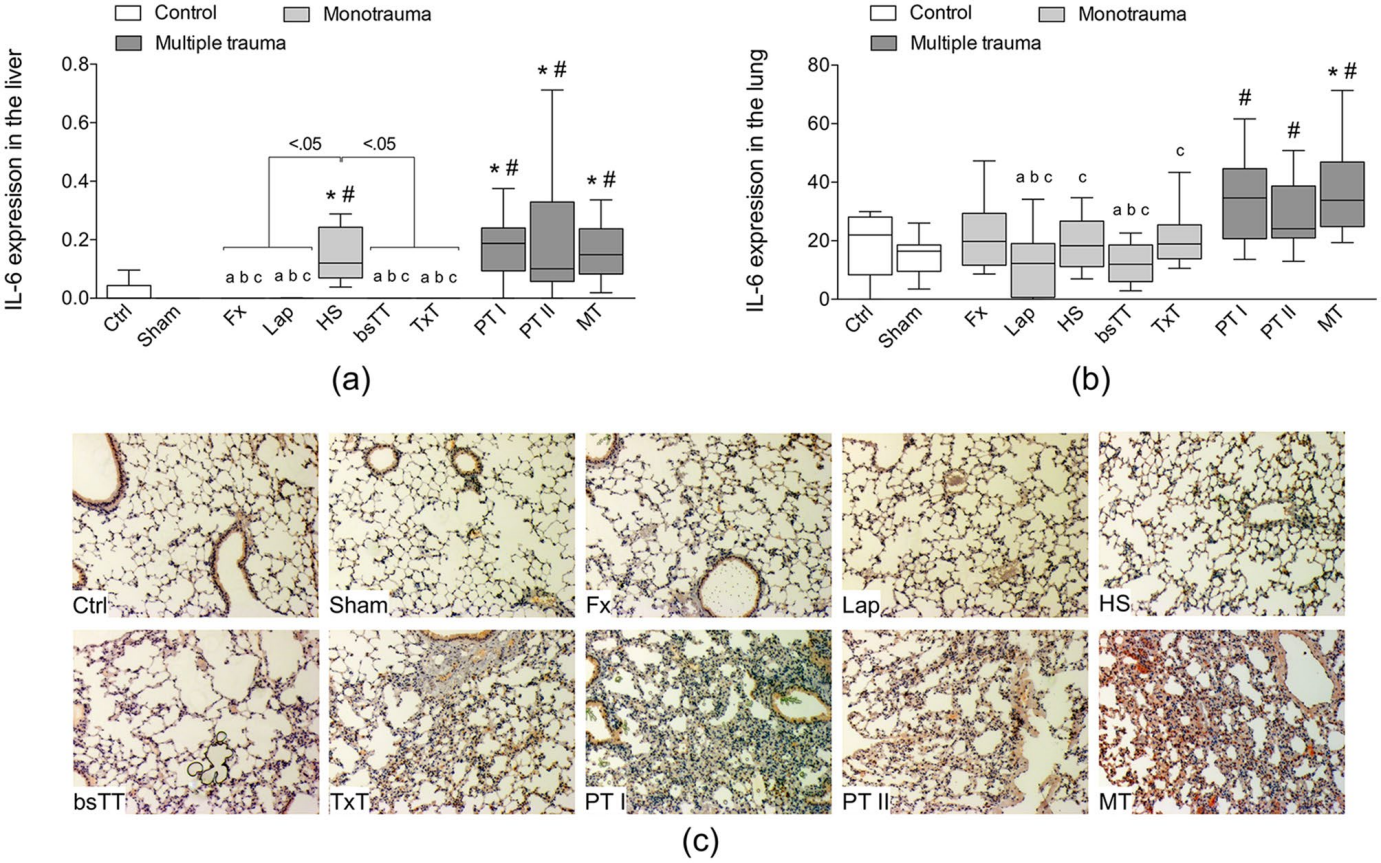
Quantitative analyses of the immune-histological staining of interleukin (IL)-6 (**a**) in the liver or (**b**) in the lung. Representative lung (**c**) sections after IL-6 staining are shown. *bsTT* bilateral soft tissue trauma, *Ctrl* no intervention, *Fx* osteotomy with external fixation, *HS* hemorrhagic shock, *Lap* midline laparotomy, *MT* multi-trauma (TxT + HS + Lap + bsTT), *PT* polytrauma (PT I: TxT + HS + Fx and PT II: TxT + HS + Fx + Lap). *Sham* surgical procedures without trauma, *TxT* thoracic trauma. Six hours after intervention, the animals were euthanized for sampling. p < 0.05 vs. indicated or * vs. Ctrl; # vs. Sham; a vs. PT I; b vs. PT II; c vs. MT.

Hepatic IL-6 expression was significantly increased in the HS group and in both polytrauma as well as in the MT group compared to Ctrl or Sham (p < 0.5, Fig. 4a). Further, the expression of IL-6 in all three multiple trauma groups was significantly enhanced compared to Fx, Lap, bsTT or TxT group (p < 0.05; Fig. 4a). Among the monotrauma groups, a significantly increased hepatic IL-6 expression in the HS group versus Fx, Lap, bsTT or TxT group was detected (p < 0.05, Fig. 4a).

Pulmonary IL-6 expression was significantly increased in both polytrauma as well as in the MT group compared to the Sham group (p < 0.5, Fig. 4b). IL-6 expression was significantly enhanced in all three multiple trauma groups compared to Lap or bsTT (p < 0.05; Fig. 4b), while HS and the TxT group, respectively, exerted only significant decrease in IL-6 expression compared to the MT group (p < 0.05, Fig. 4b). The representative histomorphological expression of pulmonary IL-6 in all experimental groups is shown in Fig. 4c.

IL-10 expression in the liver was too low for a quantification of the histological staining. However, IL-10 expression in the lung was significantly increased in all three multiple trauma groups compared to Ctrl, Sham, Fx, Lap or bsTT groups (p < 0.05, data not shown). While the HS group had significantly decreased IL-10 expression only compared to the PT I group (p < 0.05), no significant difference between the TxT group and the three multiple trauma groups was detected (data not shown).

Hepatic HMGB1 was too low for a quantification of the histological staining. However, in the lung, HMGB1 expression in the PT II group was significantly increased compared to the Ctrl or Fx group (p < 0.05, data not shown). HMGB1 expression in the MT group was significantly increased compared to the Ctrl, Sham, Fx or Lap groups (p < 0.05, data not shown). Among the monotrauma groups, the TxT group had a significantly enhanced pulmonary HMGB1 expression compared to the Fx group (p < 0.05, data not shown).

### Hepatic infiltration with neutrophilic granulocytes and liver damage

**Figure 5:**
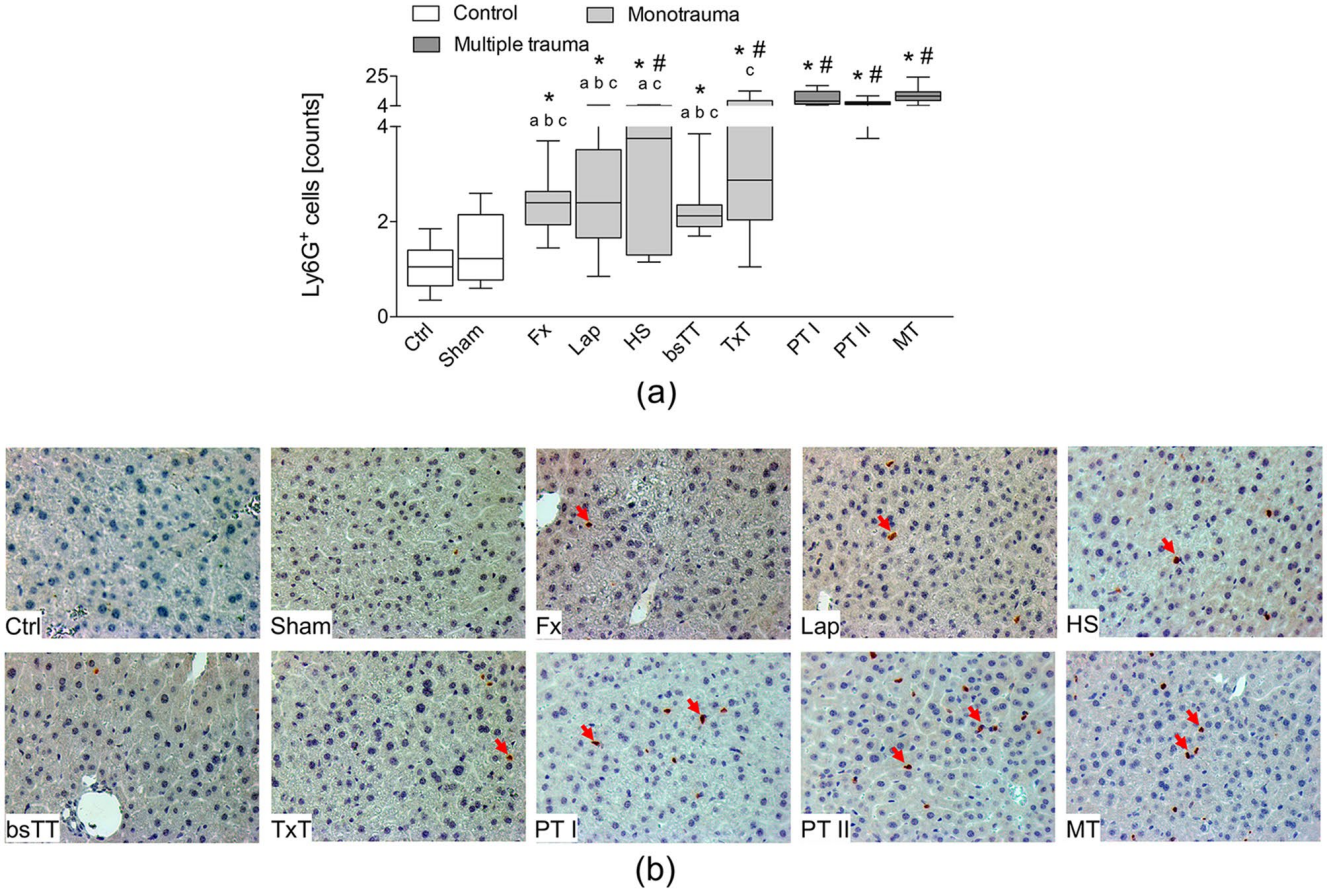
(**a**) Quantitative analysis of the immune-histological staining of Ly6G in the liver. (**b**) Representative liver sections after Ly6G staining. Positively stained cells are marked by arrows. *bsTT* bilateral soft tissue trauma, *Ctrl* no intervention, *Fx* osteotomy with external fixation, *HS* hemorrhagic shock, *Lap* midline laparotomy, *MT* multi-trauma (TxT + HS + Lap + bsTT), *PT* polytrauma (PT I: TxT + HS + Fx and PT II: TxT + HS + Fx + Lap). *Sham* surgical procedures without trauma, *TxT* thoracic trauma. Six hours after intervention, the animals were euthanized for sampling. p < 0.05 vs. indicated or * vs. Ctrl; # vs. Sham; a vs. PT I; b vs. PT II; c vs. MT.

Among all monotrauma as well as multiple trauma groups the neutrophilic infiltration into the liver significantly increased compared to the Ctrl group (p < 0.05, Fig. 5a,b). However, compared to the Sham group, the HS and the TxT as well as all three multiple trauma groups had significantly increased infiltration with inflammatory cells (p < 0.05, Fig. 5a). All three multiple trauma groups had significantly increased neutrophilic infiltration compared to the Fx, Lap or the bsTT group (p < 0.05, Fig. 5a,b). However, compared to the HS group only PT I and MT groups, while compared to the TxT group only the MT group exerted significantly higher infiltration with inflammatory cells (p < 0.05, Fig. 5a). The infiltration with inflammatory cells is associated with a trend to increased hepatic necrosis in all monotrauma and multiple trauma groups, while this trend was most prominent in HS, TxT and in all multiple trauma groups.

### Pulmonary infiltration with inflammatory cells and organ damage

The infiltration with inflammatory cells in the lung was significantly increased in all three multiple trauma groups compared to Ctrl and Sham groups (p < 0.05, data not shown). However, the infiltration with inflammatory cells among the monotrauma groups was highest in HS and TxT groups, while this trend to increased infiltration was not significant compared to control groups (data not shown).

**Figure 6:**
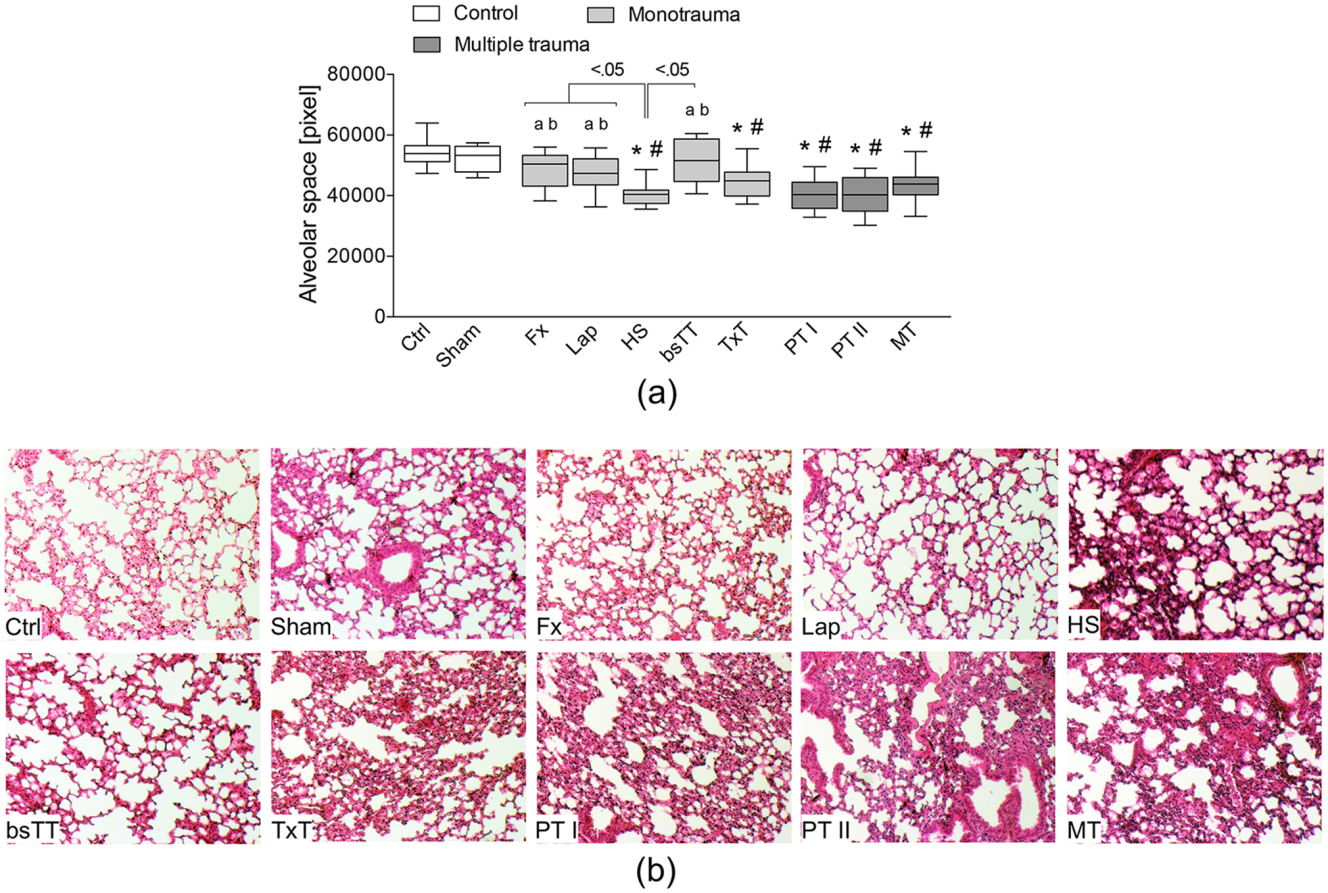
(**a**) Quantitative analysis of the alveolar space in lung sections. (**b**) Representative lung sections after hematoxylin and eosin staining. *bsTT* bilateral soft tissue trauma, *Ctrl* no intervention, *Fx* osteotomy with external fixation, *HS* hemorrhagic shock, *Lap* midline laparotomy, *MT* multi-trauma (TxT + HS + Lap + bsTT), *PT* polytrauma (PT I: TxT + HS + Fx and PT II: TxT + HS + Fx + Lap). *Sham* surgical procedures without trauma, *TxT* thoracic trauma. Six hours after intervention, the animals were euthanized for sampling. p < 0.05 vs. indicated or * vs. Ctrl; # vs. Sham; a vs. PT I; b vs. PT II; c vs. MT.

The loss in alveolar space as a sign of pulmonary damage was significant in HS and TxT as well as in all three multiple trauma groups compared to the Ctrl or Sham group, respectively (p < 0.05, Fig. 6a,b). Both polytrauma groups (PT I and PT II) had a significant loss in alveolar space as compared to the Fx, Lap or bsTT group (p < 0.05, Fig. 6a). Among the monotrauma groups, the HS group had a significant increase in loss of alveolar space compared to Fx, Lap or the bsTT group (p < 0.05, Fig. 6a). Representative lung sections with increased pulmonary damage as seen in the HS and TxT group as well as in all three multiple trauma groups compared to all other groups are shown in Fig. 6b.

## Discussion

Several attempts to develop multiple trauma models mimicking the complex post-injury situation in severely injured (polytraumatized) patients have been undertaken. Despite some promising results the suitability of those models to represent the clinical situation is still challenging and under intensive investigation. Thus, the aim of the present study was to further clarify the specific roles of diverse monotrauma as well as the relevance of the injury severity in “polytrauma” modeling. For this purpose the posttraumatic inflammatory response was evaluated in order to provide more information about a highly standardized and reproducible model which reflects the clinical situation in severely injured trauma patients.

In a recent study from our group, it was shown that both polytrauma groups as well as the multi-trauma group, which were included in this study, had an overall mortality rate of 11.7% and a profound decrease in activity^30^. Similarly, a decrease in the activity as well as a marked increase in mortality has been detected among the monotrauma groups after thoracic trauma or hemorrhagic shock^30^. Levels of the organ damage markers BUN, AST, ALT, LDH and CPK were significantly increased in PT- and MT groups compared to control, Sham or monotrauma^30^. A significant metabolic acidosis could not be demonstrated in our murine model. Only a trend towards a reduced HCO_3_^−^ concentration and a higher loss of bases could be shown for PT- and MT groups^30^. A significant compensatory decline of the pCO_2_ level was only seen in the MT group^30^.

In line with these results, we have demonstrated in the underlying study that notably the hemorrhagic shock group as well as the thoracic trauma group, on a slightly lower level, exerted clear signs of marked inflammation.

While the early mortality after trauma is mainly caused by massive blood loss, traumatic brain injuries or a non-controllable organ damage, later occurring mortality is mainly caused by secondary complications leading to (multiple) organ failure or multiple organ dysfunction syndrome (MODS)^7,31^.

A couple of experimental trauma models combining different injury severities were designed over the last decades to investigate the posttraumatic immune response after multiple trauma^9,27–30^. With regard to the clinical situation, a systemic inflammation caused by severe injuries is characteristic for the pathophysiological development of traumatized patients in the intensive care units. This inflammation appears directly after trauma and includes correlations concerning neurology, endocrinology, and hemostasis^6,7,31^. Modern estimations assume that trauma induces the systemic inflammatory response syndrome (SIRS) generated by distribution of a vast quantity of damage-associated molecular patterns (DAMPs), which relate to the entire body of the patient by systemic circulation—like high mobility group box protein 1 (HMGB1)^6,7,31,34–36^. After severe trauma, DAMPs typically induce a hyperinflammatory reaction mediated by chemokines and cytokines which is interlinked with a tenacious compensatory antiinflammatory response syndrome (CARS) leading to immunological suppression following trauma^31,36–39^. Several proinflammatory (e.g. IL-6, IL-8, CXCL family members) and anti-inflammatory (e.g. IL-10) mediators were experimentally and clinically demonstrated to play important roles in this damage response to traumatic injury, or to indicate outcomes^6,7,40,41^.

In a blunt chest trauma model, the intrapulmonary inflammatory reaction was characterized by an excessive influx of proinflammatory chemokines and cytokines including IL-6, as well as with an activation and infiltration with inflammatory neutrophils. This in turn contributed significantly to the injury of epithelial cells with subsequent capillary breakdown and microcirculatory disturbances^42–46^. Other models including chest trauma have confirmed among others the pathomechanistic importance of the proinflammatory mediators IL-6 and also CXCL1 for the development of lung damage^40,41,47,48^. Furthermore, organ injury is widely dependent on the presence of inflammatory cells in the tissue. Typically, recruitment of inflammatory cells e.g. to the lung or liver after injury involves local chemokine expression of e.g. CXCL1 or IL-6 as mentioned above. In line with these findings, the proinflammatory response to chest trauma, including infiltration with inflammatory cells, was observed both systemically and locally, while IL-6 was rather observed in the liver, CXCL1 was enhanced notably in the lungs. Taken together, there is a good concurrence in the data of those and other studies. Thus, the single chest trauma used induces a significant inflammatory response, tissue injury, and a mortality of 22% as described recently^30^, has been well established and is representing the clinically relevant and observed responses to (chest) trauma.

Hemorrhagic shock represents a major trigger of the inflammatory response after trauma, and it remains the leading cause of preventable death in trauma and follows only severe central nervous injury as a cause of mortality in this setting^49,50^. In our recent experimental study, we confirmed a significant mortality after hemorrhagic shock but as well after multiple trauma with shock^30,51^. Several traumatic multiple injury models underlined the importance of hemorrhagic shock to better reflect the human situation after polytrauma^27–29^. This was demonstrated by increased release of organ-specific transaminases as specific indicators of injury, as well as by enhanced activity loss and mortality^27,30,52^. Furthermore, IL-6 is enhanced in polytraumatized patients and predictive for the clinical outcome^53,54^. Thus, the data from the underlying study showing the highest systemic and local inflammatory changes with enhanced proinflammatory cytokines and infiltrates with inflammatory cells concurrent with organ damage in the hemorrhagic shock model, as well as in all models of multiple trauma including shock, are supporting the clinical and previous experimental reports.

The exact pathophysiological reasons for the enhanced organ injury in liver and lung in the blunt chest trauma model, hemorrhagic shock model, as well as in all three models of multiple trauma are rather speculative, however, they are concurrent with evident increase in proinflammatory cytokines and infiltration with inflammatory cells. In terms of such combinatory trauma models, only limited data exist. It is well-known that each isolated traumatic injury is survivable; however, their combination may become lethal. The development of various individual trauma models has significantly contributed to the understanding of the biological responses to injury. Yet, it remains unquestionable that further development of multiple trauma models is necessary for expanding the knowledge of the post-traumatic inflammation. In an effort to better replicate the human trauma situation in a murine model, the Injury Severity Score (ISS) greater than 15 was applied^9,27,30,55^, since an ISS > 15 is generally used as the minimum score for studies of severely injured trauma patients, as these are more likely to have poor outcomes^27,56^. Additionally, based on the term polytrauma in humans, a multiple trauma in vivo model should include three or more traumatic impacts, including life threatening injuries, such as brain, chest or abdomen injury. A few experimental models of severe (poly)trauma which fulfil those criteria have been established^27–29^. In line with those reports and above described studies, our experimental polytrauma as well as the multi-trauma model, include the two apparently most important contributing injury patterns to the post-traumatic inflammation, thoracic trauma and hemorrhagic shock. Moreover, recently, we have shown that the experimental multi-trauma model has provided comparable results to both polytrauma models with higher ISS with regard to the activity behavior, levels of transaminases as well as mortality rates^30^. Thus, further increasing of ISS may not necessarily lead to a superior model that would better represent the human trauma. Notwithstanding, we have found that all three multiple trauma models resulted in increased cytokine, chemokine and leukocyte infiltration into liver or lung. The examination of liver and lung organ damage has uncovered a marked injury in both polytrauma groups as well as in the multi-trauma group, and furthermore, it has uncovered the importance of chest trauma and hemorrhagic shock in the setting of experimental polytrauma. Thus, these findings indicate the usefulness of those multiple trauma models for studying inflammation after severe injury. Since both, systemic and local inflammation have been enhanced on the one hand, but on the other hand this increase was dependent on the evaluated parameter, it remains to be considered in future studies, that the choice of read-out parameters is also important for the appropriate model application to study therapeutic immune modulatory approaches in experimental polytrauma. We believe these models of experimental polytrauma and multi-trauma recapitulate the human inflammatory response to severe injury. Furthermore, they provide new insights into trauma modeling and also offer a new approach to evaluating therapeutic interventions aimed at alleviating the sequela of traumatic injury.

### Limitations

Our study has several limitations. The observation period of six hours after completion of trauma is too short to study organ complications and prolonged survival. Furthermore, all traumatic insults were induced within 2.5 h, while in reality traumatic injuries occur simultaneously. As already discussed on our recent study describing all models that were included here, each monotrauma model has several advantages as well as disadvantages compared to other strategies to induce the same type of injury. In this study, as well as in the previous one^30^, blunt thoracic trauma was assigned an AIS of 3, which is equivalent to a serious chest trauma with unilateral lung contusion and a rib series fracture^57^. In comparison to this an AIS of 4 is defined as severe thoracic trauma with bilateral lung contusion, rib series fracture and myocard contusion. As we could not see any rib series fracture in CT scans of our thoracic trauma model^24^, and since we have macroscopically observed at least unilateral lung contusion (data not shown), we have initially chosen to assign our blunt thoracic trauma with an AIS of 3. Yet, in the present study, the histological examination shows severe lung contusion with significant loss of alveolar space and significant pulmonary infiltration with inflammatory cells, hence, the AIS may be tossed up to 4. In this case the ISS classification as reported recently would change to following values: The TxT group would have an ISS of 16, the MT group would have an ISS of 20, the PT I group would have an ISS of 25 and the PT II group would have an ISS of 39. This remains to be considered when referring to our models that have been described recently and in this work. Unfortunately, we are not able to provide the heart rate or other organ analyses such as the kidneys and heart, which should be included in the next experimental series. The blood pressure was measured during the entire induction of trauma hemorrhage and the subsequent reperfusion, however due to ethical reasons, another measurement of the blood pressure after six hours -immediately before sacrifice—was not possible. Furthermore, we have studied a murine model, with known limitations in the mouse genome as compared to the human genome^58^.

## Conclusions

In line with our recent findings, we have shown that the experimental severe trauma model with a lower ISS has provided comparable results to both polytrauma models with higher ISS. Thus, further increasing of the ISS may not necessarily lead to a superior model that would better represent the human trauma situation. Notwithstanding, we have found that both, systemic and local inflammation have been enhanced on the one hand, but on the other hand this increase was dependent on the chosen parameters. Thus, it remains to be considered in future studies, that the read-out parameters are important for the appropriate model application to study therapeutic immune modulatory approaches in experimental polytrauma.
